# Interfacial Chemistry and the Design of Solid-Phase Nucleic Acid Hybridization Assays Using Immobilized Quantum Dots as Donors in Fluorescence Resonance Energy Transfer

**DOI:** 10.3390/s110606214

**Published:** 2011-06-09

**Authors:** W. Russ Algar, Ulrich J. Krull

**Affiliations:** Chemical Sensors Group, Department of Chemical and Physical Sciences, University of Toronto Mississauga, 3359 Mississauga Rd. North, Mississauga, ON L5L 1C6, Canada; E-Mail: russ.algar@utoronto.ca

**Keywords:** active area, biosensor, DNA, fluorescence resonance energy transfer, immobilization, regeneration, signal enhancement, quantum dots

## Abstract

The use of quantum dots (QDs) as donors in fluorescence resonance energy transfer (FRET) offer several advantages for the development of multiplexed solid-phase QD-FRET nucleic acid hybridization assays. Designs for multiplexing have been demonstrated, but important challenges remain in the optimization of these systems. In this work, we identify several strategies based on the design of interfacial chemistry for improving sensitivity, obtaining lower limits of detection (LOD) and enabling the regeneration and reuse of solid-phase QD-FRET hybridization assays. FRET-sensitized emission from acceptor dyes associated with hybridization events at immobilized QD donors provides the analytical signal in these assays. The minimization of active sensing area reduces background from QD donor PL and allows the resolution of smaller amounts of acceptor emission, thus lowering the LOD. The association of multiple acceptor dyes with each hybridization event can enhance FRET efficiency, thereby improving sensitivity. Many previous studies have used interfacial protein layers to generate selectivity; however, transient destabilization of these layers is shown to prevent efficient regeneration. To this end, we report a protein-free interfacial chemistry and demonstrate the specific detection of as little as 2 pmol of target, as well as an improved capacity for regeneration.

## 1. Introduction

Since their introduction to biological research [1,2], colloidal semiconductor quantum dots (QDs) have continued to be a promising tool in bioanalytical chemistry. QDs have broad and strong one-photon absorption, narrow and symmetric size-tunable photoluminescence (PL), and good resistance to photobleaching, making them an attractive alternative to molecular fluorophores in imaging applications and assays [3,4]. Significant interest also exits in using QDs as more than passive labels, instead opting to integrate the QD as a central component of nano-probes and biosensors [5]. In these applications, the QD serves as a scaffold for the assembly of biomolecular probes while its PL is modulated by biorecognition events. The use of fluorescence resonance energy transfer (FRET) as a mechanism of modulating QD PL is particularly advantageous and QDs are ideal donors [5–7]. The potentially high quantum yield of QDs helps maximize Förster distances, while the narrow emission can be tuned to concurrently optimize spectral overlap and reduce crosstalk between donor and acceptor channels. Furthermore, QDs can be excited over a range of wavelengths in the blue-ultraviolet region of the spectrum to allow minimization of the direct excitation of acceptors. The surface area of the QD can also be used to assemble multiple acceptors for the enhancement of FRET efficiency. Several recent reviews have highlighted bioanalyses based on QDs and FRET [5,7–9].

Our group has been interested in developing multiplexed nucleic acid diagnostics using QDs as donors in FRET. Much of this work has been based on a method for the immobilization of CdSe/ZnS QDs as a thin-film via self-assembly [10]. Thin-films of immobilized QDs are suitable for derivatization with selective interfacial biorecognition chemistry, ensure accessibility to nucleic acid targets in bulk solution, and allow the approach of chromophores at distances suitable for FRET. We developed multiplexed assays using immobilized QDs as both FRET donors and scaffolds for the immobilization of oligonucleotide probes, where the association of fluorescent acceptor dyes with sequence-specific hybridization events provided the basis for transduction. QD donors were paired with one or more acceptor dyes, and the ratios of QD PL and FRET-sensitized acceptor PL provided an analytical signal. Several different multiplexed formats were explored using different combinations of QD donors and dye acceptors: four-colour [11] and three-colour [12] two-plex assays; four- and five-colour three-plex assays [13,14]; and a six-colour four-plex assay [8,14]. Each format required consideration of spectral bandwidth occupied, materials requirements, sensitivity, the spectral resolution, and the complexity of the data analysis. Analysis times were on the order of 1–4 h, with detection limits at the nanomolar level (picomoles of target) and the ability to resolve single base pair mismatches [11]. Overall, the use of a QD-FRET strategy offers multiplexing using a single substrate, and multiple channels of analytical information are available without the need for spatial registration, imaging, sorting/single particle methods, or the use of multiple excitation sources. The method is also ensemble compatible to avoid challenges due to QD blinking, while ratiometric detection is less sensitive to variations in excitation intensity or detector sensitivity.

In this new work, we now investigate how different aspects of surface chemistry and interfacial design can be used to optimize solid-phase nucleic acid hybridization assays based on QD-FRET. Important challenges are the enhancement of sensitivity, and the development of biosensor function—*i.e.*, detection, regeneration and reuse over many cycles.

The sensitivity of ratiometric FRET measurements is limited by energy transfer efficiency and the ability to resolve small amounts of sensitized acceptor PL on a larger background of donor PL. Optimization therefore requires a reduction in donor PL background and/or an increase in sensitized acceptor PL. We present two strategies that address these two criteria individually and are based on design considerations rather than changing the intrinsic properties of the donor-acceptor pair. The first strategy is a reduction in active sensing area to reduce background donor PL; the second strategy is the use of multiple acceptors per hybridization event to increase FRET-sensitized acceptor PL. Both are generally applicable and compatible with multiplexed configurations.

Biosensor function has only been demonstrated in a limited capacity to date: a few cycles of use have been possible, but regeneration efficiency and signal magnitude have both tended to decrease with each successive cycle [11,15]. Herein, we show that this limitation has arisen from instability associated with interfacial protein layers used to improve selectivity and prevent the non-specific adsorption of nucleic acid targets on immobilized QDs. For example, the interfacial chemistry that has enabled multiplexed solid-phase QD-FRET hybridization assays is based on immobilized QDs overcoated with a layer of NeutrAvidin (NA). We present the use of a protein-free interfacial chemistry that helps to address the limitation of poor regeneration efficiency, while still maintaining good selectivity with minimal non-specific adsorption. The protein-free interfacial chemistry used a bidentate surface ligand and probe oligonucleotides modified with a terminal bidentate thiol moiety. In addition to improved regeneration, a detection limit of 2 nM (2 pmol) was obtained under conditions suitable for the discrimination of single base-pair mismatches, with hybridization times of 60 min.

**Figure 1.**
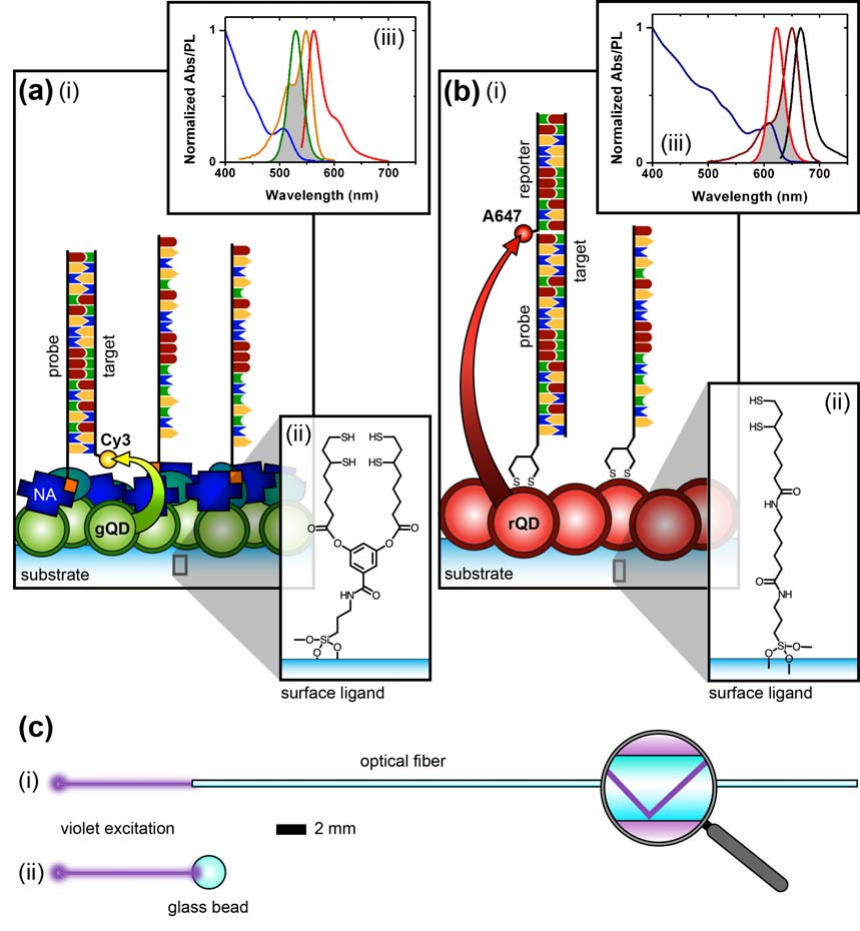
**(a)** A direct assay using protein-based interfacial chemistry: (i) immobilized QDs are coated with a layer of NA, derivatized with biotinylated probe oligonucleotides, and blocked with BSA; (ii) the nominally tetradentate surface ligand used to immobilize QDs; and (iii) spectral overlap for the gQD-Cy3 FRET pair. The gQD absorption and PL spectra are shown in blue and green, respectively. The Cy3 absorption and PL are shown in orange and red, respectively. **(b)** A sandwich assay using protein-free interfacial chemistry: (i) immobilized QDs are derivatized with dithiol-terminated probe oligonucleotides; (ii) the bidentate surface ligand used to immobilize QDs; and (iii) spectral overlap for the rQD-A647 FRET pair. The rQD absorption and PL spectra are shown in blue and red, respectively. The A647 absorption and PL spectra are shown in dark red and black, respectively. Note that the gQD-Cy3 and rQD-A647 FRET pairs can be used interchangeably with either interfacial chemistry, as can a direct or sandwich assay format. **(c)** Substrates coated with thin films of immobilized QDs and oligonucleotide probes: (i) 400 μm diameter fused silica optical fiber interrogated by total internal reflection (evanescent wave); and (ii) 2 mm dia. borosilicate glass bead interrogated in the far-field.

Figure 1 compares the NA and protein-free interfacial chemistries used in this work for hybridization assays. The former is used to investigate design criteria for optimizing sensitivity; the latter is developed to improve reusability as described above. Fused silica optical fibers and glass beads are both used as substrates. The spectral overlap for green-emitting QD-Cyanine 3 (gQD-Cy3) and red-emitting QD-Alexa Fluor 647 (rQD-A647) FRET-pairs are also shown in Figure 1, along with the direct and sandwich assay formats used in different experiments. This work clearly identifies active sensing area, the number of acceptors per hybridization event, and robust interfacial bioconjugate chemistry as important for the optimization of solid-phase QD-FRET assays.

## 2. Experimental Section

### 2.1. Reagents and Oligonucleotides

All chemical reagents were purchased from Sigma-Aldrich (Mississauga, ON, Canada) and used as received unless otherwise noted. All solvents were from Caledon Laboratories (Georgetown, ON, Canada) and were reagent grade or better. Tris-borate (TB; 100 mM, pH 7.4, 20 mM NaCl) buffer was used for all assays, and sometimes contained additives such as formamide, sodium dodecyl sulfate (SDS), or bovine serum albumin (BSA). Modified and unmodified oligonucleotides were from Integrated DNA Technologies (Coralville, IA, USA) and were HPLC purified by the manufacturer. The sequences are listed in Table 1 with abbreviations used for in-text reference. Green emitting CdSe/ZnS QDs were obtained from the Chan Laboratory at the University of Toronto. Red emitting CdSe/ZnS QD was synthesized as described previously [16]. The QDs were coated with 3-mercaptopropionic acid (MPA) using a previously used protocol [12].

### 2.2. Instrumentation

Ultraviolet-visible absorption spectra were measured using a Libra S22 spectrometer (Biochrom Ltd., Cambridge, UK) and a HP 8452A Diode-Array Spectrometer (Hewlett Packard Corporation, Palo Alto, CA, USA). Solution phase PL spectra were measured using a QuantaMaster PTI Spectrofluorimeter and Felix Software (Photon Technology International, Lawrenceville, NJ, USA). The excitation source was a xenon arc lamp (Ushio, Cypress, CA, USA) and the detector was a red-sensitive photomultiplier tube (R928P, Hamamatsu, Bridgewater, NJ, USA).

PL spectra were obtained from optical fibers using the QuantaMaster PTI Spectrofluorimeter. The fibers were illuminated by total internal reflection using a violet (407 nm) diode laser (Radius 405, 25 mW, Coherent, Santa Clara, CA, USA) and a fiber holder that was custom built to fit the sample compartment of the spectrofluorimeter [15].

**Figure 2.**
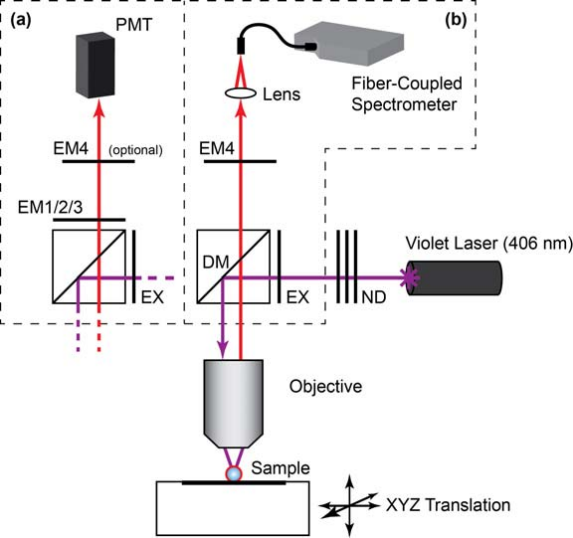
Microscope setups: **(a)** laser scanning PL imaging configuration; **(b)** PL emission spectra configuration. Refer to the text for the details of filter selection and abbreviations. The PMT in (a) can also be replaced with a monochrome CCD camera and the violet laser exchanged for a metal-halogen lamp for epi-PL imaging (not shown).

Laser scanning PL images of glass beads were obtained using a custom microscope based on a Nikon Eclipse L150 platform (Nikon Instruments Canada, Mississauga, ON, Canada). The microscope used an extra-long working distance 40× objective lens (ELWD Plan Fluor, NA = 0.60, Nikon) and a violet (406 nm) diode laser (Radius 405, 25 mW, Coherent, Santa Clara, CA, USA) that was attenuated with neutral density filters (ND). The filter cube was equipped with a *ca.* 395–415 band-pass excitation filter (EXC; z405/20×, Chroma, Rockingham, VT, USA), dichroic mirror (DM; z405rdc, Chroma), and interchangeable emission filters. The detector was a photomultiplier tube (PMT; H5784-20, Hamamatsu, Bridgewater, NJ). Samples were raster scanned under the excitation beam using a motorized xyz translation stage and data acquired using custom software written in LabVIEW (National Instruments, Austin, TX). PL spectra from glass beads were acquired by replacing the PMT with a fiber-coupled diode array spectrometer (QE65000, Ocean Optics, Dunedin, FL, USA) with Spectra Suite software (Ocean Optics). To isolate the PL from gQDs or Cy3, band-pass filters with transmission between *ca.* 515–535 nm (EM1; D525/20, Chroma) and *ca.* 560–590 nm (EM2; HQ570/20, Chroma) were used, respectively. To isolate PL from rQDs, a *ca.* 600–660 nm bandpass filter (EM3; BA 600–660, Nikon) and *ca.* 575 nm long-pass filter (EM4; FD1R, ThorLabs, Newton, NJ, USA) were used. Only EM4 was used for the acquisition of rQD and rQD-A647 PL spectra from glass beads. The combination of EM3 and EM4 was required for rQD PL imaging due to transmission of the laser line by EM3. A schematic of the instrument is shown in Figure 2.

NMR spectra were acquired using a Bruker Avance 400 MHz spectrometer (Bruker Biospin, Milton, ON, Canada). FTIR spectra were acquired using an Avatar 360 FT-IR (Thermo Scientific, Waltham, MA, USA). Electrospray ionization (ESI) mass spectra were obtained using a Waters (Milford, MA, USA) Micromass ZQ mass spectrometer.

### 2.3. Preparation of Surface Ligands

The nominally tetradentate surface ligand shown in Figure 1(a,ii) was synthesized using solid-phase methods as described previously [10,12]. The surface ligand in Figure 1(b,ii) was synthesized starting from 6,8-thioctic acid (TA, 99%), which was converted to a succinimidyl ester (TA-NHS) by reaction with 1.2 molar equivalents of *N*-hydroxysuccinimide (NHS, 98%) and 1.1 molar equivalents of diisopropylcarbodiimide (DIC, 99%) in tetrahydrofuran (THF), at room temperature over 4 h. The typical synthesis scale was 1–2 g of TA (*ca.* 5–10 mmol). The TA-NHS was isolated by crystallization from isopropanol at −18 °C overnight. The typical reaction yield was 40–60%. FTIR ν_max_ (neat): 1735/1780/1810 (C=O) cm^−1^. ^1^H-NMR (CDCl_3_) δ: 1.48–1.63 (m; 2H), 1.68–1.85 (m; 4H), 1.88–1.97 (m; 1H), 2.42–2.51 (m; 1H), 2.63 (t; 2H), 2.83 (s; 4H), 3.08–3.22 (m; 2H), 3.54–3.62 (m; 1H) ppm. ^13^C-NMR (CDCl_3_) δ: 24.33, 25.56, 28.29, 30.76, 34.39, 38.49, 40.12, 56.06, 168.38, 169.08 ppm. In a second step, 0.75–1.7 g of TA-NHS was dissolved in 35–70 mL of 5:2 methanol-pyridine, *ca.* 1.5–2 molar equivalents of 6-aminocaproic acid (ACA) were added, and the reaction was allowed to stir overnight at room temperature. The *N*-5-carboxypentyl-6,8-thioctamide (TA-ACA) product was isolated by extraction into dichloromethane using dilute hydrochloric acid as the aqueous phase, and collected by removal of the solvent under vacuum. Rapid removal of the solvent was necessary to avoid polymerization of the TA-ACA and was facilitated by the addition of a large amount of diethyl ether once the majority of the dichloromethane had been evaporated. The reaction yield was >50%. ^1^H-NMR (CDCl_3_) δ: 1.28–1.76 (m; 13H), 1.89 (m; 1H), 2.17 (t; 2H), 2.33 (t; 2H), 2.44 (m; 1H), 3.04-3.28 (m; 4H), 3.50-3.60 (m; 1H), 5.83 (s; 1H), 9.16 (s, broad; 1H) ppm. ^13^C-NMR (CDCl_3_) δ: 24.20, 25.38, 26.17, 27.79, 29.08, 33.78, 34.50, 36.37, 38.40, 39.22, 40.17, 56.36, 173.25, 178.17 ppm. ESI^−^ MS (MeOH): m/z 318.2 (M^−^).

Glass beads were cleaned and coated with 3-aminopropyltrimethoxysilane as described previously [10,11]. The TA-ACA was coupled to the aminosilanized glass beads by adding *ca.* 100–200 beads to a solution of TA-ACA (*ca.* 1.3 mmol) in THF with 1.1 molar equivalents of NHS and DIC, and letting the reaction shake overnight at room temperature. The TA-ACA-modified beads were then washed with THF, dichloromethane, diethyl ether, and stored desiccated until needed. Prior to use, the TA-ACA surface ligands were reduced to dithiolates by incubating overnight at room temperature with a 30 mM aqueous solution of tris-(2-carboxyethyl) phosphine (TCEP).

QDs were immobilized using either surface ligand in Figure 1 as described previously [10–15]. Briefly, TCEP-reduced substrates were incubated overnight in a buffered solution (pH 8.5–9.3) of MPA-coated QDs at 0.1–1.0 μM, followed by washing. The interfacial immobilization of QDs using surface ligand exchange has been characterized previously [10].

### 2.4. Synthesis of Disuccinimidyl Glutarate

Masses of 0.66 g (5 mmol) glutaric acid and 1.27 g (11 mmol) NHS were dissolved in 50 mL of anhydrous THF. A volume of 1.7 mL (11 mmol) of DIC was dissolved in a separate 15 mL volume of THF, then added portionwise to the glutaric acid and NHS solution with vigorous mixing at room temperature. The reaction was left stirring for 3–4 h, during which time a white precipitate formed. A volume of 100 mL of isopropanol was added and the precipitate dissolved. The reaction mixture was stored in the freezer (*ca.* −18 °C) for several hours. The disuccinimidyl glutarate product crystallized out of solution and was collected using vacuum filtration. The white crystals were washed with several portions of cold isopropanol, collected, and then stored in a desiccator. The reaction yield was 0.94 g (2.9 mmol, 58%). FTIR ν_max_ (neat): 1735/1785/1820 (C=O) cm^−1^. ^1^H-NMR (CDCl_3_) δ: 2.19 (m; 2H), 2.80 (t; 4H), 2.85 (s, broad; 8H) ppm. NMR ^13^C (CDCl3) δ: 19.56, 25.55, 29.56, 167.67, 168.95 ppm.

### 2.5. Assays

When using the NA-based interfacial chemistry, modified fibers or glass beads were incubated for 60 min at room temperature in buffered solutions of the desired sample oligonucleotide (Table 1) at different concentrations, followed by rinsing with buffer. In the case of sandwich assays, the sample incubation was followed by incubation for 60 min with a 150 nM (188 pmol) solution of reporter oligonucleotide containing 0.1% w/v SDS, followed by rinsing with buffer.

When using the protein-free interfacial chemistry, hybridization assays were done by shaking modified glass beads in buffered solutions of the desired sample oligonucleotide (Table 1) at different concentrations for 30–60 min. This was done at room temperature with gentle shaking, and followed by rinsing with TB buffer. In the case of sandwich assays, the sample incubation was followed by shaking with a 250 nM solution of reporter oligonucleotide for the same amount of time, followed by rinsing with TB buffer. The reporter solution sometimes contained 0.1 mg/mL of BSA.

Additional details for specific experiments are described in the Results and Discussion section. All of the assays were completed with PL measurements to determine the extent of sample hybridization, and quantitation was through calculation of a FRET ratio. Equation (1) defines the FRET ratio calculated from data obtained using the imaging configuration of the microscope (Figure 2(a)) in combination with the gQD-Cy3 FRET-pair. The subscripts denote the emission filters, and *I* denotes the PL intensity (in arbitrary units). Equation (2) defines the FRET ratio calculated for experiments that used the rQD-A647 FRET pair and spectral measurements via either the spectrofluorimeter (fibers) or the spectral configuration of the microscope (beads; Figure 2(b)).

(1) $$R={\left(\frac{I_{\text{EM}2}}{I_{\text{EM}1}}\right)}_{\text{gQD}-\text{Cy}3}-{\left(\frac{I_{\text{EM}2}}{I_{\text{EM}1}}\right)}_{\text{gQD}}$$

(2) $$R=\frac{\int I_{\text{A}647}d\lambda }{I_{\text{rQD}}d\lambda }\approx {\left(\int_{650}^{775}I_{\lambda }d\lambda /\int_{575}^{650}I_{\lambda }d_{\lambda }\right)}_{\text{rQD}+\text{A}647}-{\left(\int_{650}^{775}I_{\lambda }d\lambda /\int_{575}^{650}I_{\lambda }d_{\lambda }\right)}_{\text{rQD}}$$

## 3. Results and Discussion

### 3.1. Signal Enhancement by Minimizing Active Area

**Figure 3.**
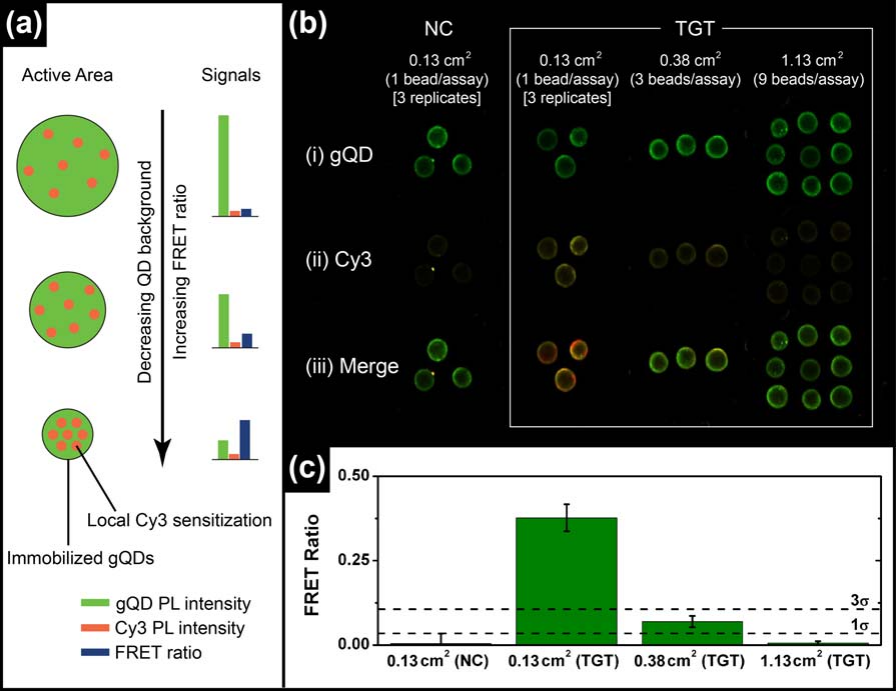
**(a)** Signal enhancement concept showing the increase in the relative amount of FRET-sensitized Cy3 PL as the gQD background is decreased. The signal strengths on the right are proportional to the areas occupied on the left. **(b)** Pseudo-colour laser scanning PL images of different numbers of glass beads used in a gQD-Cy3 direct assay to detect 10 nM (2.5 pmol) of TGT-1. **(c)** Quantitative analysis of the data in (b) that shows signal enhancement with smaller active area.

The most common format of fluorescence hybridization assay uses an interface with immobilized probe oligonucleotides. In the absence of significant non-specific adsorption, the association of fluorescent reporters with hybridization events provides an analytical signal that increases until saturation of the available probes occurs. The limit of detection (LOD) is ideally determined by the ability to resolve small increases in PL intensity on a dark background and is directly proportional to the absolute number of probe/target hybridization events, but not the degree of probe saturation. The situation is different in ratiometric QD-FRET assays, where the “background” is bright due to unquenched QD donor PL and the LOD is determined by the ability to resolve FRET-sensitized acceptor PL on that bright background. The number of donor QDs interrogated determines the magnitude of the background. Even in an ideal system, it should not be the absolute number of hybridization events that determines the magnitude of the ratiometric FRET signal, but rather the number of hybridization events relative to the number of QD donors. Since each QD is a site for the immobilization of probe(s), the signal depends on the degree of probe saturation. A potential mechanism of lowering the LOD in QD-FRET assays is therefore to decrease the active sensing area of the assay, thereby reducing the absolute number of QD donors and increasing the relative number of hybridization events (per donor) for a given concentration of target in a sample. This is illustrated conceptually in Figure 3(a).

To test the validity of the above hypothesis, solid-phase QD-FRET hybridization assays were prepared with immobilized gQD/Probe-1 (Bio) and using different numbers of glass bead substrates per sample to control the active area (*ca.* 0.13 cm^2^ per bead). Sample solutions containing 2.5 pmol of TGT-1 (10 nM, 250 μL) were agitated with one, three, or nine glass beads for 2 h at 25 °C to ensure the opportunity for nearly complete removal of the target from solution (*i.e.*, non-saturating conditions). Parallel controls with NC (Cy3) were also done. The results are shown in Figure 3(b,c) and confirm that smaller active areas facilitate the detection of smaller amounts of target. The cutoff for the limit of detection was estimated as three standard deviations above the average FRET ratio for the NC (Cy3) control samples. Only the assays that used a single bead per sample solution (0.13 cm^2^) exhibited a target signal that exceeded this threshold. The target signal for the assay with three beads per sample solution (0.38 cm^2^) was just below the LOD threshold, but more than one standard deviation higher than the average NC (Cy3) control. The assay with nine beads per sample solution (1.13 cm^2^) had no apparent target signal above the NC (Cy3) control. Previous assays on optical fibers had an active area of *ca.* 38 mm^2^ and a LOD of 1.3 pmol (1 nM in 1.25 mL) [11]. Linearly extrapolating to an assay with a 0.01 mm^2^ active area (e.g., 100 × 100 μm pad or 113 μm diameter spot) suggests that the anticipated LOD can reach *ca.* 0.3 fmol (300 fM in 1.25 mL) or better in microanalytical systems. Active areas with this order of dimensionality can be readily achieved in microfluidic systems, which can also offer advantages such as small sample sizes, dynamic control of stringency and rapid hybridization kinetics [17]. Microfluidic chips are thus well suited as a potential platform for solid-phase QD-FRET nucleic acid hybridization assays.

### 3.2. Signal Enhancement Using Multiple Acceptors

**Figure 4.**
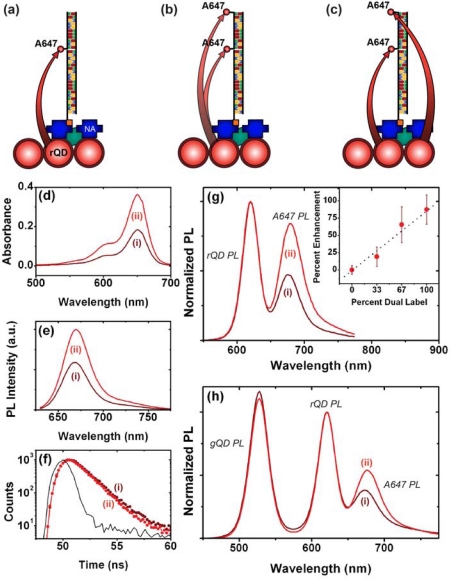
**(a)** A single energy transfer pathway using REP-2 (A647). **(b)** Two possible non-concurrent energy transfer pathways that could occur with REP-2 (2× A647). **(c)** Two possible concurrent energy transfer pathways that could occur with REP-2 (2× A647). **(d)** UV-visible absorption and **(e)** fluorescence spectra for equal concentrations of (i) REP-2 (A647) and (ii) REP-2 (2× A647). **(f)** Fluorescence decay profiles for (i) REP-2 (A647) and (ii) REP-2 (2× A647). Signal enhancements from the replacement of (i) REP-2 (A647) with (ii) REP-2 (2× A647) in **(g)** one-plex and **(h)** two-plex formats. The inset in (g) shows a gradual enhancement with an increasing relative amount of REP-2 (2× A647).

As noted earlier, one of the advantages of using QDs as donors in FRET is the ability to array multiple acceptors around each QD (multivalency) in order to improve FRET efficiency. The interfacial configurations in Figure 1 do not provide this opportunity directly. Nonetheless, the association of multiple acceptor dyes with each interfacial hybridization event is expected to provide an enhancement of FRET efficiency and an increase in the magnitude of FRET-sensitized acceptor PL. It is estimated that QDs can immobilize at densities approaching 10^13^ cm^−2^ [10]. In contrast, studies have shown that oligonucleotide probes tend to immobilize at densities closer to 10^12^ cm^−2^ [18–20], suggesting that immobilized QD donors may be in excess of the number of acceptors even at saturation of hybridization of the immobilized probes. Figure 4(a–c) illustrates the potential mechanisms through which FRET-sensitized acceptor PL can be enhanced in an excess donor configuration. When a single acceptor dye is associated with a probe/target hybridization event, it is proximal to multiple QD donors. However, given that experiments have shown that typical acceptor dye PL lifetimes are not dramatically shorter than immobilized QD donor lifetimes [21], there is only a small probability that more than one of those donors will transfer its energy to one particular proximal acceptor during the average excited state lifetime of the system. The association of a second acceptor dye with a unique hybridization event offers two mechanisms of enhancement: first, each proximal QD can interact with two acceptor dyes, increasing the FRET efficiency in a manner analogous to the multivalency effect in bulk solution; second, the proximal QDs that did not sensitize the first acceptor dye still have the opportunity to sensitize the second acceptor dye.

A fiber-based sandwich assay was designed to evaluate the potential for signal enhancement using multiple acceptors per hybridization event. A reporter oligonucleotide, REP-2 (2× A647), with acceptor dyes at both its 3′ and 5′ termini, was used in combination with immobilized rQDs and Probe-2 (Bio). As shown in Figure 4(d–f), REP-2 (2× A647) has the optical properties that would be expected *a priori*: it has approximately twice the absorption and fluorescent intensity of an equal concentration of REP-2 (A647), and the fluorescence lifetime of the A647 is largely unchanged. To test the enhancement in a one-plex format, a sandwich assay was done with immobilized rQDs/Probe-2 (Bio), incubation with 100 nM (125 pmol) TGT-2 (NL), and exposure to different reporter solutions. The reporter solutions contained a fixed total concentration of reporter oligonucleotide, but increasing amounts of REP-2 (2× A647) relative to REP-2 (A647). Figure 4(g) shows that a linear increase in FRET ratio was observed with increasing REP-2 (2× A647), and yielded an average of >80% signal enhancement when REP-2 (2× A647) was used exclusively. The observation of a linear increase is consistent with a greater number of QD donors than dye acceptors. The observation that REP-2 (2× A647) does not quite double the FRET signal can be attributed to the larger rQD donor-3′A647 acceptor separation—and intrinsically lower FRET efficiency—compared to the 5′A647 acceptor. To test the enhancement in a two-plex format, the experiment was repeated using a mixture of co-immobilized gQDs and rQDs. In this case, the use of REP-2 (2× A647) provided a 40–50% signal enhancement (Figure 4(h)) compared to the use of REP-2 (A647). The smaller enhancement relative to the one-plex format is due to the substitution of immobilized rQD donors with gQDs that are not spectrally resonant with the A647 acceptor. Overall, the results clearly demonstrate that the association of multiple acceptor dyes with each hybridization event can offer signal enhancements in solid-phase QD-FRET assays. Future work will investigate the extent to which this effect can be further amplified by incorporating one or more internal labels along the length of a reporter oligonucleotide, or periodically along the length of another oligomer (e.g., peptide, dendrimer) that is ligated to a reporter oligonucleotide.

### 3.3. Selectivity and Regeneration with Protein-Based Interfacial Chemistry

One of the most common methods of controlling stringency in nucleic acid hybridization assays is the use of a temperature that lies between the melt temperatures (*T*_m_) of the fully complementary target and, for example, a single base-pair mismatch [22]. Temperature was a variable initially explored in QD-FRET assays, but met with little success, likely due to the increased lability of physisorbed protein layers and QD ligands at elevated temperatures (>50 °C) [15]. A more successful alternative was the use of formamide as a chemical denaturant, which has the effect of lowering *T*_m_ by approximately 0.6 °C for each percent (v/v) added [23], thus enabling high stringency at close to room temperature. For example, we previously found that 25% v/v formamide provided stringency that was sufficient to suppress hybridization of Probe-1 with 1BPM-1 while still permitting hybridization of TGT-1 [11]. However, finding conditions for efficient assay regeneration was more difficult. Neither harsh conditions such as 85% formamide at 25 °C, nor milder conditions such as 30–50% formamide between 25–35 °C, were effective [11,15]. In every case, the regeneration efficiency degraded with each cycle, and the amount of signal decreased in each successive round of hybridization. Additives such as BSA and SDS were also ineffective at improving regeneration efficiency, despite enhancing selectivity under assay conditions. However, a remarkable observation was that none of the regeneration conditions compromised the ability of the NA-based interfacial chemistry to resist the non-specific adsorption of NC sequences under assay conditions (see Figure 8(a–b) for examples) [11,15]. This enigmatic result suggested that the interfacial dynamics were not straightforward, and motivated the work presented herein.

**Figure 5.**
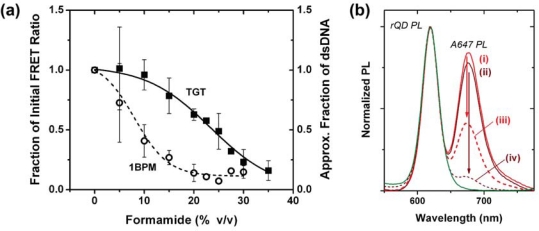
**(a)** Formamide denaturation profiles for Probe-1/TGT-1 and Probe-1/1BPM-1 hybrids. **(b)** Comparison demonstrating the effect of DSG-crosslinking on the NA-based interfacial chemistry: FRET signal in response to 150 nM (188 pmol) TGT-2 (A647) for interfaces (i) without and (ii) with DSG crosslinking; and the decrease in the FRET signal after regenerating using 70% formamide at 25 °C for interfaces (iii) without and (iv) with DSG crosslinking. The rQD PL spectrum is shown for reference.

One hypothesis for the poor regeneration efficiency of the NA-based interfacial chemistry was that the mild conditions were insufficient to completely denature Probe-1/TGT-1 hybrids, and that selectivity under assays conditions was the consequence of a mixture of kinetic and thermodynamic effects. Although the calculated *T*_m_ of the Probe-1/TGT-1 hybrids was 23.1–11.1 °C in the presence of 30–50% formamide, it is known that immobilization of oligonucleotide probes at an interface can cause shifts in the value of *T*_m_ and alter the steepness of the melt transition [24,25]. To this end, a full chemical denaturation profile was measured for the Probe-1/TGT-1 hybrids at 25 °C by setting up a series of sandwich hybridization assays with incremental increases in formamide concentration. The stability of the Probe-1/1BPM-1 hybrid was investigated similarly to also evaluate selectivity, and the two profiles are shown in Figure 5(a). The results confirm that a formamide concentration of 20–25% provides optimal selectivity between TGT-1 and 1BPM-1 (*vide infra*). Considering regeneration, the profile for the denaturation of TGT-1 is broad and may require ≥35% formamide for complete denaturation at 25 °C. Nonetheless, the range of conditions previously tested appear to be sufficient for denaturation of Probe-1/TGT-1 hybrids, thus suggesting that poor regeneration efficiency was due to another aspect of the interfacial chemistry.

To further characterize the effects of regeneration conditions, a direct hybridization assay on fibers with immobilized rQDs/Probe-2 (Bio) was carried out at two sets of relatively harsh conditions where it was known that TGT-2 would not hybridize and the degree of non-specific adsorption was of interest. At 25 °C and with 70% formamide, the FRET ratios obtained for TGT-2 (A647) and NC (A647) were 0.06–0.08 and statistically indistinguishable, indicating no hybridization and a small amount non-specific adsorption. At 45 °C and with 40% formamide, the FRET ratios obtained were 0.4–0.9 and were also statistically indistinguishable, but indicated a large amount of non-specific adsorption. It had been hoped that the ability to reduce the amount of formamide necessary to melt the Probe-1/TGT-1 hybrid by using a higher temperature would be advantageous; however, this latter result suggested that temperature was potentially important in activating the NA-based interfacial chemistry toward non-specific adsorption.

The prior observations that non-specific adsorption occurred under regeneration conditions, but not *after* exposure to regeneration conditions [11,15], suggested transient destabilization of the protein layer of the interfacial chemistry. In this work, transient destabilization was confirmed by incubating fibers modified with either Probe-2 (Bio) or Probe-3 (Bio) in 200 nM (250 pmol) of TGT-2 (A647) under assay conditions. The FRET ratio from fibers modified with Probe-2 was *ca.* 2.0, while that from fibers modified with Probe-3 was effectively nil. The same fibers were then incubated in the same volume of 40% formamide at 45 °C for 90 min and measured again. The fibers modified with Probe-2 were poorly regenerated with a FRET ratio of 0.81. More importantly, the fibers modified with non-complementary Probe-3 exhibited an increase in signal to a FRET ratio of 0.04. This was due to the non-selective adsorption of TGT-2 that dehybridized from fibers that were modified with Probe-2, and subsequently diffused to fibers modified with Probe-3 where it adsorbed. Thus, the regeneration conditions transiently destabilized the protein layer that had blocked non-selective adsorption under hybridization conditions. The high initial interfacial concentration of hybridized target upon exposure to regeneration conditions is expected to promote adsorption and, although probe-target hybrids are denatured, this adsorption prevents efficient regeneration and reuse. The observation that temperature has a large effect is consistent with an activation energy barrier for reorganization of the surface that is driven, at least in part, by the presence of a high concentration of formamide. The mechanism of action of formamide on the *T*_m_ of nucleic acids is through the disruption of hydrogen bonds [26], and formamide is likely to have a similar effect on proteins.

Given the success of the NA-based interfacial chemistry in hybridization assays, it was desirable to explore methods for stabilization of the protein layer against the action of formamide—in particular, chemical crosslinking. The first method tested was NA-modified (no probe) fiber exposure to a solution of amine-reactive 1% w/v glutaraldehyde for between 10–30 min after deposition of the NA layer on immobilized QDs. This was followed by immobilization of the biotinylated probes. Unfortunately, glutaraldehyde crosslinking decreased hybridization signals in direct assays by as much as 35% (data not shown). Avidin differs from NA in that it has three glucosamine residues (and other carbohydrates) per subunit, potentially providing a greater capacity for glutaraldehyde crosslinking. However, even larger decreases in hybridization signal were associated with crosslinking of the Avidin (>50%), suggesting interference with the biotin-binding site of the Avidin and NA. This was confirmed experimentally using Cy3 labeled Probe-1 (Bio), where glutaraldehyde crosslinking could cause up to a 30% decrease in the efficiency of probe oligonucleotide immobilization (data not shown). To address this challenge, disuccinimidyl glutarate (DSG) was adopted as a structurally analogous amine-reactive crosslinker, but one that had different reactivity than glutaraldehyde. While glutaraldehyde can crosslink nucleic acids, the reaction of DSG with nucleobases is much slower and poorly competes with hydrolysis and reaction with primary amines (e.g., lysine residues). This allows probe to be immobilized prior to crosslinking. Fibers modified with the full NA-based interfacial chemistry using rQDs/Probe-2 (Bio) and were incubated with a 1.8 mg mL^−1^ aqueous solution of DSG (18% DMSO) for 30 min. The fibers were then exposed to samples with 150 nM TGT-2 (A647) for 60 min, the PL spectrum measured, incubated with 70% formamide at 25 °C, and PL measured again. A parallel experiment was run with non-crosslinked fibers and the corresponding PL spectra are shown in Figure 5(b). The FRET ratios associated with DSG crosslinked fibers decreased from 1.36 after sample exposure, to 0.12 after regeneration. FRET ratios associated with non-crosslinked fibers decreased from 1.51 to 0.71. This represented 91% regeneration efficiency with DSG crosslinking compared to 53% regeneration efficiency without. In contrast to the glutaraldehyde, the DSG crosslinking decreased the initial hybridization signal by only 10%. While this further confirmed that the transient destabilization of interfacial protein layers was limiting the capacity for regeneration, the regeneration efficiency with DSG crosslinking was still insufficient for practical use. This was further compounded by poor recovery of hybridization signals in a subsequent cycle of use. The results suggest that stabilization of NA layer may not be directly related to the stability and extent of hybridization.

### 3.4. Development of Protein-Free Interfacial Chemistry and Assays

**Figure 6.**
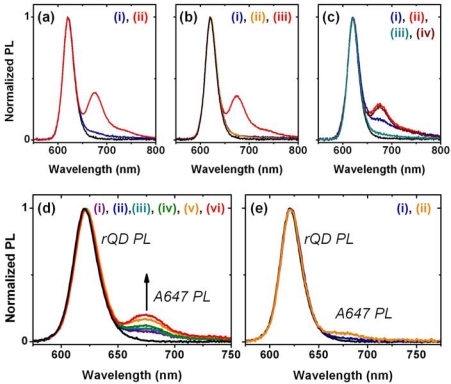
**(a)** Pseudo-colour laser scanning PL images of (i) a TA-ACA modified glass bead without exposure to rQDs, (ii) TA-ACA modified beads after incubation with rQDs, and (iii) TCEP-reduced TA-ACA modified beads after incubation with rQDs. The scale bar is 2 mm. **(b)** Pseudo-colour epi-PL image of an rQD-modified bead from (iii). The scale bar is 0.5 mm and the apparent halo in PL intensity is due to curvature of the bead relative to the focal plane. **(c)** Comparison of the QD PL spectrum between bulk solution and immobilized *via* the reduced TA-ACA surface ligand.

The results described above indicate that the protein layers used in previous designs of solid-phase QD-FRET nucleic acid hybridization assays are potentially limiting with respect to regeneration and reuse. Moreover, when using a NeutrAvidin bridge to derivatize immobilized QDs with oligonucleotide probes, the protein dimensions (*ca*. 6.0 × 5.5 × 4.0 nm [27]) add distance to the overall separation between the QDs and acceptor dyes, thereby reducing energy transfer efficiency. A protein-free strategy thus has the potential to improve both sensitivity and reusability. In parallel with developing protein-free interfacial chemistry, we explored the use of a new TA-ACA surface ligand (Figure 1(b,ii)) for QD immobilization. The new ligand replaces potentially labile ester bonds with stable amide bonds, and avoids the need for multi-step solid-phase synthesis following the aminosilanization of substrates. To confirm that the TA-ACA surface ligand was effective for the immobilization of QDs, ligand-modified glass beads were incubated with an aqueous solution of rQDs, both with and without TCEP reduction, and characterized using PL measurements. Figure 6(a) shows that reduction of the TA-ACA disulfide to thiol groups was necessary for efficient QD immobilization. The average PL contrast between QDs immobilized on beads with the disulfide- and thiol-terminated surface ligands was 1:4, suggesting that coordination of the thiolate to the ZnS shell of the QDs was the primary driving force behind immobilization. The QD PL intensity obtained from immobilization was homogeneous across the surface of the bead (Figure 6(b)). Comparing the QD PL spectra between bulk solution and on the bead surface, a small bathochromic shift (*ca.* 1 nm) and increase in full-width-at-half-maximum (*ca.* 1 nm) were observed after immobilization (Figure 6(c)). This was consistent with previous results obtained using the tetradentate surface ligand (Figure 1(a,ii)) for QD immobilization [10]. The immobilization of QDs at a surface creates close proximity between individual QDs, and the changes in PL spectra can be attributed to energy transfer from smaller QDs (*i.e.*, hypsochromic edge of the PL spectrum) to larger QDs (*i.e.*, bathochromic edge) within the distribution of sizes across the population [28].

In order to implement protein-free derivatization of the immobilized rQDs with oligonucleotide probes, Probe-1 (SS) was designed with a 5′ disulfide that could be reduced with TCEP and directly conjugated to the QDs *via* self-assembly. While a previous study used a six-carbon alkyl thiolate linker to conjugate probe oligonucleotides to immobilized QDs, no selectivity could be achieved without the use of BSA as a blocking agent [15]. This result led us to develop the NA-based interfacial chemistry. In the current work, the minimal length and mobility of the linkage associated with disulfide modification (Figure 1(b)) of the 5′ terminus of the probe oligonucleotides, as well as the tighter binding of QD by the dithiol compared to a single thiol, suggested the possibility of forming a more dense and ordered interfacial layer that could better resist non-specific adsorption. Support for this hypothesis was found in previous studies with QD-oligonucleotide conjugates in bulk solution, where increased loading of the QD with bound oligonucleotides decreased the ability of other nucleic acids in solution to adsorb [29,30]. Although the incubation of the immobilized QDs with an excess of dithiol-modified probe could potentially cause displacement of the surface ligands by competing for binding sites on the QD shell, this was not observed in the experiments. The surface ligands were expected to coordinate to the underside of the immobilized QDs, and the interfacial density of QDs likely isolated the layer of surface ligands from the probes in bulk solution.

**Figure 7.**
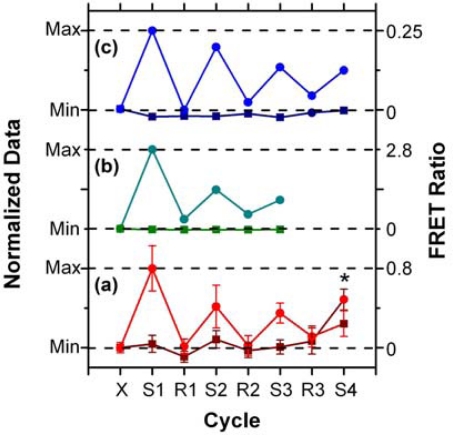
**(a)** Direct assay and response to: (i) 100 nM NC (A647) and (ii) 100 nM TGT-1 (A647). **(b)** Direct assay with 25% v/v formamide and response to: (i) 100 nM NC (A647), (ii) 100 nM 1BPM-1 (A647), and (iii) 100 nM TGT-1 (A647). **(c)** Sandwich assay and response to: (i) 100 nM NC (NL); (ii) 100 nM TGT-1 (NL); (iii) 100 nM NC (NL) with 0.1% BSA in the reporter solution; and (iv) 100 nM TGT-1 (NL) with 0.1% BSA in the reporter solution. Reporter solutions were 250 nM REP-1 (A647). **(d)** Sandwich assay with 25% formamide in sample solutions that contained: (i) 2 nM, (ii) 5 nM, (iii) 10 nM, (iv) 20 nM, (v) 40 nM, or (vi) 80 nM of TGT-1 (NL). The reporter solution contained 0.1% BSA and 250 nM REP-1 (A647). **(e)** The same sandwich assay as in (d), showing response to 100 nM NC (NL) and 1BPM-1 (NL). The initial rQD PL spectrum is shown for reference in each case.

Assays were done in different formats and under different conditions to evaluate the utility of the protein-free interfacial chemistry. In each case, immobilized rQDs were derivatized with Probe-1 (SS). Figure 7(a) shows the results of a direct assay in aqueous buffer solution, where a FRET ratio of 0.61 was obtained for 100 nM (100 pmol) of TGT-1 (A647) and had better than 7:1 contrast over an equal amount of NC (A647) sequence. This observation confirmed the hypothesis that derivatization of the QDs with dithiol-modified probe oligonucleotides would offer better selectivity than that previously obtained with alkyl thiolate-modified probes (negligible contrast). The sequence associated with TGT-1 is diagnostic of the neuromuscular disease spinal muscular atrophy, but requires the ability to distinguish TGT-1 from a single nucleotide polymorphism [22]. The second test of the interfacial chemistry was thus a direct assay that incorporated 25% v/v formamide to evaluate the potential for single base-pair mismatch discrimination. As shown in Figure 7(b), the assay selectivity was improved, where 100 nM (100 pmol) of TGT-1 (A647) yielded a FRET ratio of 0.62 and contrast of 8:1 over both NC (A647) and 1BPM-1 (A647) sequences. The final proof-of-concept test of the interfacial chemistry was a sandwich assay, which is of more practical interest than a direct assay since it enables the use of unlabeled targets. Modified beads were first incubated with a solution of sample nucleic acids, then exposed to a solution of reporter oligonucleotide, REP-1 (A647). Figure 7(c) shows that when REP-1 (A647) was introduced, there was substantial non-selective adsorption and a false positive signal. The contrast between TGT-1 (NL) and NC (NL) was only 1.5:1. However, the addition of 0.1 mg mL^−1^ of BSA to the reporter solution greatly improved the selectivity, increasing the contrast to almost 6:1. The BSA had minimal affect on the TGT-1 (NL) signal, with the FRET ratio decreasing to 0.43 from 0.48 without BSA. The greater non-specific adsorption of the 15-mer REP-1 (A647) compared to the 19-mer TGT-1 (A647) is consistent with results previously obtained using the NA-based interfacial chemistry [11], and may be indicative of a size-threshold below which oligonucleotides can efficiently permeate the layer of immobilized probes and adsorb within interstitial spaces between immobilized QDs. The development of the protein-free interfacial chemistry allows use of BSA an additive to the reporter solution rather than as an integral component of the interfacial chemistry or additive in the sample solution.

The data in Figure 7(a–c) indicated that optimum selectivity could be achieved with 25% v/v formamide in the sample solution and 0.1 mg mL^−1^ BSA in the reporter solution. Figure 7(d) shows increasing FRET-sensitized A647 PL corresponding with increases in the amount (2–80 nM; 2–80 pmol) of TGT-1 (NL), and demonstrates the potential for quantitative analysis. Figure 7(e) shows the response to 100 nM (100 pmol) of NC (NL) and 1BPM-1 (NL) sequences under the same conditions. The contrast between target and a single base-pair mismatch was such that 2 nM of the former gave a 40% larger FRET signal than 100 nM of the latter. The LOD in previous sandwich assays based on optical fibers with an equivalent active area and NA-based interfacial chemistry was 1 nM (1.3 pmol) [11]. This required a 4 h hybridization period due to the absence of convective mixing, and was also obtained in the absence of formamide. The ≤2 nM (2 pmol) LOD obtained in this study—despite working at 25% formamide with a *ca.* 50% loss in TGT-1 binding (Figure 5(a))—is attributed to the shorter donor-acceptor separation distance that is possible in the absence of a NA layer between immobilized QDs and probe oligonucleotides. This may also offset a potential decrease in QD quantum yield (decreases the rate of FRET) due to the loss of extra passivation from the protein layer [31,32]. The shorter hybridization time (60 min) is attributed to the use convective mixing with bead substrates. The limiting factor for the LOD was not the signal magnitude, but rather the spectral noise inherent to the microscope used for measurements, suggesting the potential for a sub-nanomolar (sub-picomole) LOD.

### 3.5. Regeneration and Reuse with Protein-Free Interfacial Chemistry

**Figure 8.**
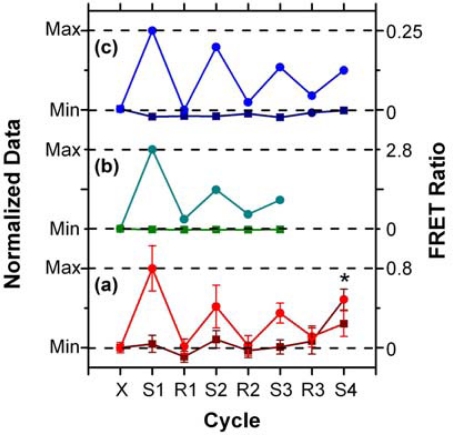
Regeneration and reuse of different interfacial chemistries in direct assay formats: **(a)** the protein-free interfacial chemistry (7 M urea, 25 °C); **(b)** NA-based interfacial chemistry (30% formamide, 35 °C, 0.1% SDS, 0.1 mg/mL BSA); and **(c)** chemistry based on oligonucleotide probes terminated with a monodentate thiol linker and an interfacial layer of BSA (85% formamide, 25 °C). The data in (b) and (c) are taken from previous studies [11,15]. Dark coloured lines are for NC samples; light coloured lines are for TGT samples. The asterisk (*) indicates that TGT and NC samples were switched. Abbreviations: X, initial; S, sample exposure; R, regeneration cycle.

In addition to supporting an effective hybridization assay, the primary objective of developing the protein-free interfacial chemistry was an improvement in assay reusability. This was tested in a direct assay format to monitor the probe/target interaction without convolution from the two-step hybridization of reporter oligonucleotides in sandwich assays. Initially, regeneration was attempted using 50% formamide at 25 °C; however, this was observed to deleterious effect on the QD PL intensity. This effect was also observed previously with immobilized QDs modified with monodentate thiol-terminated oligonucleotide probes and could be somewhat reversed in completely aqueous solution [15]. Nonetheless, we opted to investigate the use of urea as an alternative regeneration strategy in the current work. The urea acts similarly to the formamide, effectively decreasing the *T*_m_ of the probe-1/TGT-1 hybrids below 25 °C at a 7 M concentration, but was not used previously with the NA-based interfacial chemistry due its well-known ability to efficiently denature proteins [33]. As shown in Figure 8(a), the protein-free interfacial chemistry showed no statistically significant response to NC (A647) over three cycles each of use and regeneration. Considering TGT-1 (A647) hybridization, 30 min with 7 M urea at 25 °C was able to efficiently regenerate the interfacial chemistry to a level that was indistinguishable form the NC (A647) samples within the precision of the experiment. In contrast, previous study has found inefficient regeneration of both NA-based interfacial chemistry [11], and also chemistry that used a monodenate thiol-linker for probe oligonucleotide with an interfacial BSA layer [15]. Figure 8(b,c) show that the regeneration of these interfacial chemistries was characterized by divergence between the baseline FRET ratios of the NC and TGT samples, as well as a gradual decrease in the FRET ratio with each hybridization cycle. In the current work, an initial drop was observed, but the FRET ratio may have stabilized afterward. Statistically, the second and third hybridization steps (cycles 3 and 5) have similar FRET ratios, as does the fourth (cycle 7), where the beads used in previous cycles as controls for NC (A647) were exposed to TGT-1 (A647), and *vice versa*. Although the reason for the initial drop is currently unclear, we hypothesize that it may be due, at least in part, to reorganization of the oligonucleotide probes under denaturating conditions that could disrupt inter-probe or probe-QD interactions. The observation of this trend, as well as resistance to non-specific adsorption over multiple cycles with the different interfacial chemistries studied to date, suggests that the diminishing signal is unlikely to be due to the loss of immobilized probe with successive cycles. Overall, the results indicate that robust interfacial chemistry is best suited for efficient regeneration and reuse of solid-phase QD-FRET hybridization assays. Future work will investigate methods for the covalent derivatization and stabilization of immobilized QDs.

## 4. Conclusions

Assays and biosensors based on the use of QD donors in FRET offer several advantages for multiplexing and microanalytical systems. In this study, we have identified several different strategies for the optimization of solid-phase nucleic acid hybridization assays based on QD-FRET, which are all based on the design of interfacial chemistry. That is, they can be generally applied with any QD donor-dye acceptor FRET pair. The LOD in assays can be improved by reducing QD donor PL background through minimization of the active sensing area. It is anticipated that the incorporation of QD-FRET assays in microanalytical systems could yield LODs that are orders of magnitude better than those observed with macroscopic fiber and bead substrates. Sensitivity can also be improved by associating multiple acceptor dyes with each hybridization event. This takes advantage of the excess of immobilized QD donors, and can potentially be scaled beyond two acceptor dyes per hybridization event. In addition, we developed a protein-free interfacial chemistry for QD-FRET hybridization assays, demonstrating that it was suitable for quantitative detection to 2 nM (2 pmol) or less of target, with the discrimination of single base pair mismatches. Importantly, this interfacial chemistry also offered improved capacity for regeneration and reuse when compared to protein-based interfacial chemistries. Transient destabilization of the NA-based interfacial chemistry under regeneration conditions was identified as the limitation to obtaining good regeneration in previous studies. When combined with the minimization of active area and the use of reporters labeled with multiple acceptors, the further development of robust interfacial chemistries with compact linkages between immobilized QDs and oligonucleotide probes will provide the opportunity for reusable QD-FRET biosensors that offer excellent sensitivity and high throughput.

## Figures and Tables

**Table 1.** Oligonucleotide sequences.

***Probes***	

Probe-1 (Bio)	**Biotin**-5′-ATT TTG TCT GAA ACC CTG T-3′
Probe-1 (SS)	**S_2_C_4_H_7_**-5′-ATT TTG TCT GAA ACC CTG T-3′
Probe-2 (Bio)	**Biotin**-5′-CTT ACT TCC ATG ATT TCT TTA ACT-3′
Probe-3 (Bio)	**Biotin**-5′-AAC AAT ATT GTC TTG ATT-3′

***Targets***	

TGT-1 (NL)	3′-TAA AAC AGA CTT TGG GAC ATT CCT TTT ATT TCC T-5′
TGT-1 (Cy3)	**Cy3**-3′-TAA AAC AGA CTT TGG GAC A-5′
TGT-1 (A647)	3′-TAA AAC AGA CTT TGG GAC A-5′-**A647**
1BPM-1 (NL)	3′-TAA AAC ACA CTT TGG GAC ATT CCT TTT ATT TCC T-5′
1BPM-1 (Cy3)	**Cy3**-3′-TAA AAC ACA CTT TGG GAC A-5′
1BPM-1 (A647)	**A647**-3′-TAA AAC ACA CTT TGG GAC A-5′
TGT-2 (A647)	**A647**-3′-GAA TGA AGG TAC TAA AGA AAT TGA-5′
TGT-2 (NL)	3′-GAA TGA AGG TAC TAA AGA AAT TGA TGC GGC CCT AGG TAG-5′

***Reporters***	

REP-1 (Cy3)	**Cy3**-5′-AA GGA AAA TAA AGG A-3′
REP-1 (A647)	**A647**-5′-AA GGA AAA TAA AGG A-3′
REP-2 (A647)	**A647**-5′-ACG CCG GGA TCC ATC-3′
REP-2 (2×A647)	**A647**-5′-ACG CCG GGA TCC ATC-3′-**A647**

***Non-complementary***	

NC (Cy3)	3′-TGT CCC AAA GTC TGT TTT A-5′-**Cy3**
NC (A647)	3′-TCA ATT TCT TTA GTA CCT TCA TTC-5′-**A647**
NC (NL)	3′-AGG AAA TAA AAG GAA TGT CCC AAA GTC TGT TTT A-5′
